# Can competition improve hospital quality of care? A difference-in-differences approach to evaluate the effect of increasing quality transparency on hospital quality

**DOI:** 10.1007/s10198-021-01423-9

**Published:** 2022-01-08

**Authors:** Christoph Strumann, Alexander Geissler, Reinhard Busse, Christoph Pross

**Affiliations:** 1 Institute of Family Medicine, University Hospital Schleswig-Holstein, Campus Luebeck, Luebeck, Germany; 2 School of Medicine, University of St. Gallen, St. Gallen, Switzerland; 3 Department of Health Care Management, Berlin University of Technology, Berlin, Germany

**Keywords:** Hospital quality of care, Hospital competition, Public reporting, Difference-in-differences, I11, D24

## Abstract

Public reporting on the quality of care is intended to guide patients to the provider with the highest quality and to stimulate a fair competition on quality. We apply a difference-in-differences design to test whether hospital quality has improved more in markets that are more competitive after the first public release of performance data in Germany in 2008. Panel data from 947 hospitals from 2006 to 2010 are used. Due to the high complexity of the treatment of stroke patients, we approximate general hospital quality by the 30-day risk-adjusted mortality rate for stroke treatment. Market structure is measured (comparatively) by the Herfindahl–Hirschman index (HHI) and by the number of hospitals in the relevant market. Predicted market shares based on exogenous variables only are used to compute the HHI to allow a causal interpretation of the reform effect. A homogenous positive effect of competition on quality of care is found. This effect is mainly driven by the response of non-profit hospitals that have a narrow range of services and private for-profit hospitals with a medium range of services. The results highlight the relevance of outcome transparency to enhance hospital quality competition.

**Supplementary Information:**

The online version contains supplementary material available at 10.1007/s10198-021-01423-9.

## Introduction

Most developed countries are facing a steady increase of hospital expenditures, which account for the majority of total health-care costs [1]. Several reforms have been implemented, all to increase hospital efficiency, e.g., the introduction of activity-related budgets steered by diagnosis-related groups (DRG)-based payment systems. To assess and prevent potential negative consequences of the increased cost pressure on hospital quality [2, 3], the importance of quality in managerial decision and policymaking is increasingly taking a central role. Substantial quality variations and treatment differences suggest an enormous potential for quality improvement [4–6].

Recent legislative efforts in several countries have advanced public reporting (PR) on quality of care with the intention to inform health system stakeholders and guide patients to find the provider with the highest quality [7]. In Germany, PR has been implemented in 2008 for all acute care hospitals [8]. Annual, self-reported hospital data are compiled as part of the mandatory external quality monitoring system gathering structural information on inpatient numbers, diagnoses, procedures and risk-adjusted quality indicators [9]. Several transparency portals report this information in a publicly accessible and patient-friendly manner, e.g., Weisse Liste.de (WL.de). Most studies analyzing the effects of PR suggest that health-care providers are stimulated to improve quality [10, 11]. In Germany in particular, indicators have been deemed reliable [12] and consumers regard quality information as useful to differentiate better from worse hospitals [13]. However, as concluded by Metcalfe et al. [14], the existing evidence base is insufficient to derive general policy recommendations on how to further develop PR initiatives. One reason for this might be that the economic mechanism how PR should result in general health-care quality improvements is often not considered: besides empowering patients to choose the hospital with the highest quality, another aim of PR is to foster a fair competition for the best quality. As suggested by economic theory, competition and information are complementary tools for promoting health-care quality [15], if patients’ choice of hospital is influenced by quality [16], as e.g., shown by Kuklinski et al. [17] and Emmert et al. [18] for patients in Germany.

In most developed countries, health-care markets are highly price regulated enabling hospitals only to compete for customers on non-price dimensions such as quality [19]. Hospitals will increase quality to gain market share as long as price is above marginal costs [20]. This concept has been tested empirically primarily in US and UK health-care markets with ambiguous results [21]. The results depend heavily on the degree of price regulation between providers, the transparency of quality of care and the mobility of patients [22, 23]. With a slight consensus toward a positive effect of competition on quality of care in a fixed price setting, empirical results are mixed and vary across indicators, treatment areas and study designs [24, 25]. Regarding the effect of PR, it has been shown that the stronger the competition, the stronger is the quality improvement after quality disclosure [26, 27].

In Germany, hospitals provide primarily inpatient treatment and services. The hospital landscape is characterized by substantial overcapacities controlled by state governments, a uniform DRG-based payment system, freedom of choice for patients about their provider and a considerable heterogeneity between hospitals [28–30]. For instance, hospitals differ in their ownership type (public, non-profit, and private for-profit) and the range of services provided. The introduction of quality transparency should encourage hospitals to invest higher efforts in quality improvement [31, 32]. Hospitals compete with each other; their different ownership types and treatment varieties might determine their flexibility and resources to maintain or advance their competitive position. For many years, all hospitals have been confronted with a reduction of the legally obliged public capital investment support, forcing hospitals to invest independently. Private for-profit hospitals have generally better access to private capital than public or non-profit hospitals that are not allowed to accumulate profits except for reinvestments [33]. As a result, the investment rate of private for-profit hospitals is higher in comparison with the rate of public and non-profit hospitals [34]. Further, private hospitals receive more often payments for newly approved technologies that are negotiated between individual hospitals and health insurances [35]. Results based on Swiss hospital data show that production uncertainties due to unpredictable variability of demand differ between private for-profit and public hospitals as well as along the degree of specialization [36]. Hospitals that are planning their resources based on expected future demand face a trade-off between the probability of undersupply and excess capacity [37, 38]. The results of Widmer et al. [36] show that Swiss private specialized hospitals are able to predict most accurately the future demand, because of their specialization on few, mostly elective procedures. As a result, they can more easily avoid excess capacities. Instead, hospitals providing a broad variety of treatments to ensure regional population health coverage are encouraged to avoid situations of undersupply.

The aim of this study is to examine whether increased competitive pressures through the introduction of PR has improved the quality of hospital care in Germany. Since hospitals are expected to increase quality under fixed prices to gain market share with different means, a homogenous reform effect is questionable. To test for this possible heterogeneity, we estimate specific effects for distinct types of hospitals that differ in ownership and degree of specialization. We apply a difference-in-differences (DiD) design since the effect of the public release of performance data through PR portals in 2008 is expected to be higher in markets that are more competitive [26, 27]. In these markets, hospitals are assumed to be at a higher risk to lose patients to their competitors if quality differences in favor of neighboring hospitals can be observed publicly [39, 40]. Panel data from 947 hospitals for 2006 and 2010 are used. Overall hospital quality of care is measured by the 30-day mortality rate for stroke treatment, adjusted for patient characteristics, comorbidities and used procedures. Stroke mortality is considered as a good proxy for the overall quality of the hospital due to the high complexity of stroke care, established care protocols and the interdisciplinary nature of the treatment of stroke patients [5]. Quality improvement in emergency stroke care requires structural and procedural advancements (i.e., stroke unit) and continuous improvement cycles via quality management system. Compared to in-hospital mortality, data on 30-day mortality has the advantage of measuring quality comprehensively as it does not favor hospitals with short length of stays [41]. Moreover, in contrast to other hospital quality measures and disease areas, 30-day mortality for acute care for stroke patients is characterized by high variation [6, 42], which allows for a better differentiation between hospitals’ quality and, thus, provides more statistical power. Market structure is measured by two complementary approaches: (1) the Herfindahl–Hirschman index (HHI) based on market shares of stroke patients and (2) the number of hospitals providing stroke care in the respective hospital’s market. The latter measure is expected to be exogenous and not affected by the hospital quality (at least in the short run), since in Germany, despite overcapacities closing hospitals is a rather difficult and long-lasting process [43]. For the former measure, however, endogeneity cannot be ruled out. To allow a causal interpretation of the reform effect based on the HHI, we follow Gaynor et al. [39] and Cooper et al. [40], and substitute actual market structure with predicted market shares that are based on exogenous variables only.

This paper expands the literature along three primary angles. First and foremost, we expand the research on the effects of hospital competition on quality of care, in particular in the German market context where this research is scarce [44]. Further, our data allows us to integrate outcomes 30 days after hospital discharge. This measure addresses the incentive of hospitals to discharge or transfer a patient to a rehab facility as early as possible. Moreover, the treatment of stroke represents a complex emergency care situation, which can capture the overall organizational choice of quality of care to be provided and efficiency in diagnostic and therapeutic provision of care [5]. Second, we are the first in Germany to use the introduction of hospital quality transparency as an intervention in DiD empirical framework and, thus, are able to identify causal effects. Third, we provide further empirical evidence on the hospital market structure in Germany, which is generally scarce [45].

## Data and empirical implementation

### Data

We have assembled a comprehensive hospital-level panel dataset, derived from clinical, administrative and regulatory data sources on a variety of structural and outcome quality measures at the hospital level as well as demographics at the district level. We integrate data from two premier hospital quality reporting systems in Germany: the QSR (Qualitätssicherung mit Routinedaten) outcome indicators from Germany’s largest sickness fund, the Allgemeine Ortskrankenkasse (AOK), and the mandatory hospital quality monitoring system of the Federal Joint Committee (G-BA) that are only available every second year starting from 2006. The patient-based (risk-adjusted) QSR outcome indicators for stroke are centrally calculated by the WIdO research institute (Wissenschaftliches Institut der AOK) and are based on administrative data of AOK-insured patients. The quality indicators are used from 2006 and 2010. Hospitals with less than 50 beds are excluded from the analyses due to disproportional high fluctuation and low data quality [46] resulting in a panel data of 947 hospitals.

### Policy intervention for difference-in-differences design

Under a DRG-based payment system with fixed prices within a region and unrestricted patient choice, hospitals compete on quality to attract patients [24]. However, due to high information asymmetries between patients and physicians, patients often have limited opportunities to assess treatment options, process of care, and their respective prospects for success in advance. In Germany, PR for all acute hospitals has become mandatory in 2004 and effective in 2008. Annual, self-reported hospital data are compiled as part of the mandatory external quality monitoring system. It gathers structural information on inpatient numbers, diagnoses and procedures. Furthermore, process, outcome and risk-adjusted outcome quality indicators for 30 diseases and diagnoses, covering around 3.1 million cases or 15% of the annual case volume in Germany are provided [9]. The transparency portal Weisse Liste.de (WL.de) carries out the government mandate to publicly report the information in an easily accessible and patient-friendly manner. A study analyzing the usage behavior of WL.de between 2013 and 2015 revealed that the number of daily users was 1445 in 2013, making 28 visits per 1000 hospital admissions. Until 2015 the number of daily users has increased by 38% to 2753 (52 visits per 1000 hospital admissions) [8]. While WL.de is the leading German portal, other initiatives such as Qualitätskliniken.de also offer online quality of care information for participating hospitals. In summary, the 2004 reform resulted in several PR portals launching in 2008, with quality of care and its shortcomings topping the health policy agenda and public discussion [47].

In a non-price competitive environment, transparency about quality of hospital care promotes the mobility of patients to choose the hospital with the highest quality and, thus, is intended to encourage hospitals to invest higher efforts in quality improvement to attract patients [31, 32]. The policy intervention with increased quality transparency has led to uncertainties about potentials and consequences for hospitals [48], which face different strategic, financial and operational options to respond and improve their quality. Consequently, a heterogeneous response to the introduction of quality transparency can be expected.

### Measurements

#### Hospital quality

Examining quality of care for the entire hospital or jointly for several medical conditions can be problematic given condition-specific risk and resource profiles [49]. Therefore, we focus on a specific medical indication and use a risk-adjusted outcome measure. We consider the quality of care in stroke treatment, since (1) stroke patients show often a range of severe comorbidities which are treated by interdisciplinary teams and departments within a hospital [5, 50] and (2) treatment protocols are well established. Furthermore, stroke is a frequent medical condition with a high outcome variation across hospitals [6].

Our measure of hospital quality is the 30-day risk-adjusted standardized mortality rate (SMR) that includes events up to 30 days after hospital admittance. It is defined as the ratio of the number of observed and expected mortality. The latter is predicted by means of a logistic regression of patient attributes such as age, gender and comorbidities (diabetes, hypertension, etc.) on mortality [51]. The risk adjustment ensures comparability of outcomes between different hospitals and their respective patient samples. Moreover, including post-discharge data allows us to control for the possibility that due to the incentive structure of the DRG-based prospective payment system, hospitals discharge patients too early.

#### Measures of market structure

The market structure is measured by two complementary approaches. First, based on the stroke case volumes in each hospital, we calculate the market share of hospitals performing stroke treatment and the HHI for each hospital market. In our empirical analysis, we focus on the HHI as it is a simple and robust competition measure. As a second and complementary measure of the market structure, we also consider the number of hospitals providing stroke care in the respective hospital’s market.

With regard to the geographical market, we employ a geographic radius of 15 km around each hospital´s geo-location. Patients travel on average 18 km to their hospital and the third quartile travel distance was 31 km in 2014 [52]. The number of hospital competitors indicated by Hentschker et al. [45] (8.5) is very close to the number of competitors in this study in the 15 km radius instance (7.8–8.0) (Table 1). In emergency care such as stroke, travel distances are also often shorter than in elective disease areas such as joint replacement. In flexible radius methods, however, related studies often include the districts that add up to 80% of the patient population and a 30 km radius is also an often chosen distance [53]. Therefore, as a robustness check, we vary our radius geographic market definition and also include 30 km radius.

A causal interpretation of the estimated effect of competition on hospital quality requires exogeneity of the market structure variable. The number of hospitals is highly regulated and under jurisdiction of state and local governments, thus slow to respond to market changes and seem not to be affected by quality performance in the short run [43]. Therefore, we consider the number of hospitals as an exogenous measure of the market structure. Regarding HHI, based on patient flows and the market from which the hospital draws large share of its patients (e.g., 80%), it might be prone to endogeneity. High-quality hospitals might attract more patients leading to higher market shares [39]. Furthermore, patients attracted to higher-quality hospitals might have a higher severity level, which can bias both outcome measures (if risk adjustment is incomplete) and market shares. This leads to potential reverse causality, with the regressor variable HHI depending on the dependent quality variable.

To prevent this endogeneity problem when using HHI as a measure for the market structure, we instrument actual patient flows with an exogenous measure [22, 23, 39, 40]. Instead of using the actual number of stroke patients to compute the HHI, we predict the number of stroke patients by means of applying a negative binomial model for count data based on exogenous factors. The predicted number of patients is used to compute the market shares for generating the (exogenous) HHI. As explanatory variables for the predicted number of patients, we utilize hospital characteristics (ownership, service status, teaching, university status, and size), regional characteristics (federal state dummy variables, the district’s urbanization level, population density, physician density, unemployment, population health and age) and year effects. All these variables are not affected by the quality of a specific hospital or unobserved patient heterogeneity and, thus, can be considered as exogenous. For instance, in Germany the capacity of beds is determined by the states for most hospitals and therefore an exogenous factor for the hospitals at least in the short run [54, 55]. However, one might suggest that hospitals apply for additional beds if they can indicate sufficient patient numbers. To reduce this (potential) effect, we use wide categories of beds (i.e., 0–150 beds, 151–300 beds, 301–600, more than 600) as a measure for hospital size instead of the number of beds.

#### Other controls

Observed and unobserved time-invariant differences between hospitals as variations in the hospitals’ case mixes are considered by hospital fixed effects. Many potential control variables are suspected to be endogenous (e.g., admissions of stroke patients, staff levels). That is why we use a rather limited number of controls. This includes the wide categories of beds as an exogenously determined measure of hospital size and variables on the district level, such as the regional mortality rate, average age of the population, the unemployment rate and the gross domestic product per capita (GDP). The district level variables are included to approximate variations in the health status of the population. All control variables are measured in natural logarithms to account for unequal variation.

### Empirical implementation

We use hospitals as the unit of analysis and measure market structure at the hospital level^1^ for a geographically fixed radius. To test the hypothesis that the increased transparency between hospitals improved hospital quality, we examine whether quality is lower or higher for hospitals in highly or less concentrated markets after an increase of competition due to the policy reform by means of a DiD approach. Since all hospitals are affected by the reform at the same time, we follow Gaynor et al. [39] and Cooper et al. [23], and apply a continuous treatment approach. The intensity of the competition induced by the reform depends on the market structure and, thus, the latter can serve as a continuous treatment variable. Hospitals located in less concentrated markets face more potential competitors and, thus, are more affected by an increase of competition induced by the policy reform, than hospitals located in highly concentrated markets with less choice for patients (or the emergency response service).

The analysis is focused on a short window (2 years before and 2 years after) around the effective implementation of PR portals in 2008 to avoid contamination from earlier or later policy changes, such as the introduction of the DRG-based payment system in 2004 [56]. Moreover, this narrow and symmetric time window around the introduction of the reform reduces the potential bias due to a violated parallel trend assumption [57]. Explicitly, we choose 2006 as the baseline period, which is compared with data from 2010, 2 years after the policy change. The DiD specification reads as

1 $${y}_{it}= {\beta }_{1}I\left(t={T}_{1}\right)+ {\beta }_{2}I\left(t={T}_{1}\right)\times {MS}_{i,t={T}_{0}}+{\beta }_{3}\times {\mathrm{MS}}_{it}+ {{\varvec{x}}}_{it}^{^{\prime}}{\beta }_{4}+ {\mu }_{i}+{e}_{it},$$

where ${y}_{it}$ is the outcome variable, i.e., the standardized mortality rate of hospital $i$ at time $t$, and $I\left(\bullet \right)$ is an indicator function, taking the value 1 for the post-reform year ${T}_{1}$ and 0 otherwise. The market structure is measured by ${\mathrm{MS}}_{it}\in \{{\mathrm{HHI}}_{it},{\widehat{\mathrm{HHI}}}_{it},{\#\mathrm{hosp}}_{it}\}$, with ${\mathrm{HHI}}_{it}$, ${\widehat{\mathrm{HHI}}}_{it}$ and ${\#\mathrm{hosp}}_{it}$ denoting the actual HHI, the predicted HHI and the number of hospitals in the market, respectively. The market structure of the baseline year ${T}_{0}$ is denoted as ${\mathrm{MS}}_{i,t={T}_{0}}$. The effect of the policy reform is given by the DiD coefficient ${\beta }_{2}$. The post-reform year effect ${\beta }_{1}$ controls for any common macro changes including a potential global (or isolated from the market structure) effect of PR on hospital quality. Observable hospital and regional characteristics which vary over time are collected by ${{\varvec{x}}}_{it}^{^{\prime}}$. Time-invariant heterogeneity among the hospitals is considered by the fixed effects ${\mu }_{i}$. Finally, ${e}_{it}$ denotes disturbances that are allowed to be heteroskedastic and correlated arbitrarily over time.

To allow for subgroup-specific DiD coefficients, Model (1) is extended by

2 $$\begin{aligned} y_{it} & = \beta_{1}^{1} I\left( {t = T_{1} } \right) \times G_{i}^{1} + \cdots + \beta_{1}^{P} I\left( {t = T_{1} } \right) \times G_{i}^{P} \\ & \quad + \beta_{2}^{1} I\left( {t = T_{1} } \right) \times {\text{MS}}_{{i,t = T_{0} }} \times G_{i}^{1} + \cdots + \beta_{2}^{P} I\left( {t = T_{1} } \right) \times {\text{MS}}_{{i,t = T_{0} }} \times G_{i}^{P} \\ & \quad + \beta_{3}^{1} \times {\text{MS}}_{it} \times G_{i}^{1} + \cdots + \beta_{3}^{P} \times {\text{MS}}_{it} \times G_{i}^{P} + {\varvec{x}}_{it}^{^{\prime}} \beta_{4} + \mu_{i} + e_{it} , \\ \end{aligned}$$

where ${G}_{i}^{P}$ is assigned a value of 1 if the $i$-th hospital belongs the $p$-th group with $p\in \{1,\dots , P\}$ in the baseline year ${T}_{0}$ and 0 otherwise.

Firstly, to estimate different effects for subgroups of hospitals accordingly to their degree of specialization, we differentiate between highly, medium and non-specialized hospitals. A hospital is identified as highly specialized if the degree of specialization ($\mathrm{spec}$) is within the third tercile (> 66%) of the sample. Accordingly, we consider hospitals as low specialized if $\mathrm{spec}$ is below the first tercile (< 33%). Medium specialized hospitals have a degree of specialization which is within the second tercile (33–66%). The degree of specialization is measured by an information theory index in terms of differences between the national and the hospital’s proportions of cases belonging to several diagnosis categories [58, 59]. Let ${p}_{ij}$ be the proportion of treated patients in diagnosis category $j$ of hospital $i$ and ${\theta }_{j}$ the national average of proportions. The information theory index is then given by

$${\text{spec}}_{i} = \sum\limits_{j = 1}^{J} {p_{ij} \ln \left( {\frac{{p_{ij} }}{{\theta_{j} }}} \right)} .$$

If the hospital’s proportions of cases belonging to several diagnosis categories are equal to national proportions, there is no specialization (${\mathrm{spec}}_{i}=0$). The index increases with increasing differences between the hospital's proportions and nationwide proportions.

Secondly, the type of ownership (public, non-profit and for-profit) determines the considered subgroups. Thirdly, we combine both classifications and specify different effects for highly specialized public, medium specialized public, non-specialized public hospitals and so forth.

To justify that the DiD coefficients identify the causal effect of the reform on quality, we need to assume parallel trends in hospital quality prior to the policy reform. Since we only have data for one pre-intervention period, i.e., 2006, we cannot graphically inspect whether hospital quality has developed parallel for hospitals located in markets with different competition levels. Instead, we examine if quality in 2006 of hospitals of distinct competition groups (high, medium, low) do vary significantly in levels. If there are no significant differences, we assume that the same mechanism that had resulted in this outcome would imply parallel trends.

## Results

We first present descriptive statistics, followed by the test result of the parallel trend assumption and our regression results, differentiating between a homogenous and a heterogeneous treatment effect. We also show the results of additional robustness checks to ensure validity and sustainability of the policy reform effects.

### Descriptive analysis

We first examine the data patterns for our competition and outcome quality measure. Table 1 displays the means for all variables for the considered baseline and post-reform year across the subgroups considered for estimating the heterogenous treatment effects. The balanced panel sample consists of 947 hospitals, which represent 66% of the total case volume in Germany. The average number of treated cases increased by 11% from 2006 to 2010. For stroke cases, an increase of 7.5% over time can be observed. Hospital quality improved from 2006 to 2010. Stroke care has been slightly more concentrated between 2006 and 2010, which is in line with the policy measures to increasingly treat patients in specialized stroke units. Predicted HHIs tend to be lower than actual HHIs, although they are highly correlated (0.87, not shown in the table). This highlights the potential endogeneity of the HHI based on actual patient flows, which seem to be determined by potentially endogenous factors such as unobserved hospital quality.

**Table 1:** Descriptive statistics

	Year	All	Specialization			Ownership		
			Low	Med	High	Non-profit	Private for-profit	Public
Sample characteristics								
Number of stroke hospitals								
All	2006	947	315	316	316	450	134	363
	2010	947	315	316	316	447	150	350
Non-profit	Both	897	191	362	344	–	–	–
Private for-profit	Both	284	71	71	142	–	–	–
Public	Both	713	368	199	146	–	–	–
Number of total cases per hospital	2006	12,510	18,789	11,530	7231	10,320	10,199	16,079
	2010	13,768	20,660	12,665	8001	11,303	11,003	18,102
Number of stroke cases per hospital	2006	183.9	283.3	141.3	127.4	132.5	175.9	250.5
	2010	212.2	336.1	155.7	145.4	140.9	190.6	312.6
Hospital quality								
30 days standardized mortality rate	2006	1.09	1.15	1.13	0.99	1.13	0.99	1.08
	2010	1.04	1.09	1.13	0.91	0.99	1.06	1.10
Competition measure for 15 km								
Actual HHI: ${\mathrm{HHI}}_{it}$	2006	0.48	0.56	0.46	0.43	0.38	0.55	0.57
	2010	0.52	0.60	0.51	0.46	0.43	0.57	0.61
Predicted HHI:${\widehat{\mathrm{HHI}}}_{it}$	2006	0.39	0.47	0.37	0.34	0.30	0.47	0.48
	2010	0.40	0.48	0.37	0.33	0.31	0.46	0.48
Number of hospitals in a market:${\#\mathrm{hosp}}_{it}$	2006	7.8	5.9	8.2	9.4	10.1	6.2	5.7
	2010	8.0	6.0	8.4	9.6	10.4	6.4	5.8
Control variables								
Beds (50–150)	Both	0.21	0.04	0.20	0.39	0.21	0.34	0.16
Beds (151–300)	Both	0.33	0.27	0.41	0.31	0.38	0.28	0.28
Beds (301–600)	Both	0.33	0.44	0.30	0.24	0.35	0.27	0.32
Beds (> 600)	Both	0.13	0.24	0.09	0.06	0.05	0.11	0.23
Log(mortality)	Both	2.32	2.33	2.30	2.32	2.32	2.33	2.30
Log(age)	Both	3.66	3.67	3.66	3.67	3.66	3.67	3.66
Log(unemployment)	Both	2.09	2.08	2.08	2.12	2.19	2.07	1.97
Log(GDP)	Both	3.40	3.37	3.40	3.42	3.41	3.31	3.41

### Parallel trend assumption

In Table 2, the mean and standard deviation of the hospital quality measure and the market structure variables are denoted for different groups of hospitals located in markets characterized by high, medium and low competition. While in low competitive markets, there are, on average, 1.5 hospitals, high competitive market consists of 17.4 hospitals. Although there is some variation in hospital quality across the distinct groups, estimated coefficients of a regression of stroke mortality on respective group dummy variables indicating low, medium, and high competition are not (jointly) significant. Assuming that the same mechanism that have resulted to this outcome also operated in previous years, parallel trends can be derived that justify the identification of the causal effect of PR.

**Table 2:** Testing for outcome differences

Market structure variable	Competition	Market structure		Stroke mortality		Regression analysis^a^	
		Mean	Std. Dev.	Mean	Std. Dev.	Coefficient	*F* test^b^
${\mathrm{HHI}}_{it}$	Low (*n* = 315)	0.86	0.15	1.10	0.55	Reference	
	Med (*n* = 316)	0.43	0.09	1.04	0.69	− 0.062	1.46
	High (*n* = 316)	0.15	0.05	1.12	0.64	0.019	
${\widehat{\mathrm{HHI}}}_{it}$	Low (*n* = 315)	0.73	0.21	1.06	0.51	Reference	
	Med (*n* = 316)	0.32	0.08	1.09	0.68	0.029	1.03
	High (*n* = 316)	0.12	0.04	1.13	0.69	0.071	
${\#\mathrm{hosp}}_{it}$	Low (*n* = 293)	1.5	0.5	1.06	0.52	Reference	
	Med (*n* = 337)	4.3	1.4	1.08	0.70	0.018	0.72
	High (*n* = 317)	17.4	6.8	1.12	0.63	0.059	

Based on 947 hospitals with 2006 data. A hospital is located in a market with low competition if the HHI is within the third tercile (> 66%) of the sample. Accordingly, we consider markets as highly competitive if HHI is below the first tercile (< 33%). Medium competitive markets have an HHI which is within the second tercile (33–66%). For the number of hospitals in a market, we consider the first tercile (< 33%) and third tercile (> 66%) to mark low and highly competitive markets, respectively

^a^Regressing stroke mortality on group dummy variables indicating low, medium, and high competition, respectively

^b^*F* test statistic with 2 and 944 degrees of freedom for joint significance of the group dummy variables

### Regression results

The estimated effects of the DiD specification are shown in Tables 3, 4 and 5. The regression models control for year effects, hospital size, regional factors and hospital fixed effects. In each table, the results of separate models for the three measures of the market structure to capture treatment intensity are shown.

In all model specifications, neither the hospital size nor the regional factors have a significant effect on the quality of care, except the unemployment rate which shows a significant effect (at the 10% level) in some models. This might be due to the fixed effects specification that considers all the observed and unobserved time-invariant differences between hospitals as variations in the hospitals’ case mixes.

#### Homogenous treatment effect

In Table 3, the estimation results of the DiD specification of a homogenous treatment effect are shown. All models obtain a significant DiD coefficient. Considering the models 1 and 2, the positive DiD effects suggest that after the formal introduction of quality transparency in 2008, mortality decreased more quickly in markets that are more competitive. The same interpretation holds for the negative DiD effect if the market structure is measured by the number of hospitals in the market; the introduction of PR has a stronger negative effect in markets with more hospitals on mortality. The difference between the DiD effect of Model 1 (${\mathrm{HHI}}_{it}$) and Model 2 (${\widehat{\mathrm{HHI}}}_{it}$) indicates that the competition effect is slightly overestimated if endogeneity is not taken into account.

**Table 3:** Fixed effects difference-in-differences estimates of the direct effects of the policy reform on stroke mortality

	HHI		Number of hospitals
	Actual	Predicted	
	(${\mathrm{HHI}}_{it}$)	(${\widehat{\mathrm{HHI}}}_{it}$)	(${\#\mathrm{hosp}}_{it}$)
Model	1	2	3
Postref-year *2010*	− 0.15	− 0.09	0.24
Market structure	0.76**	0.13	0.02
DiD: 2010 × market structure	0.35**	0.27**	− 0.02***
Controls			
Beds (50–150)	− 0.10	− 0.11	− 0.12
Beds (151–300)	0.04	0.06	0.03
Beds (301–600)	0.05	0.03	0.03
Reference (> 600)			
Log(mortality)	− 0.95	− 0.86	− 0.99
Log(age)	3.41	3.63	0.55
Log(unemployment)	0.45	0.36	0.53*
Log(GDP)	0.33	0.32	0.60
Observations	1894	1894	1894
LOGLIKE	− 1252.0	− 1259.0	− 1249.9
AIC	2524.0	2537.9	2519.8

Significance levels: ***1%; **5%; *10%

#### Heterogenous treatment effects

In the following, we relax the assumption of a homogenous treatment effect and decompose the policy reform effect to different (more homogenous) subgroups of hospitals. In Table 4, the estimation results of the separated DiD effects are shown. All models show an increase in the model fit in comparison with the homogenous effect models (1–3), as indicated by the Akaike Information Criterion (AIC). In all models, substantial differences in the reform effects between the subgroups are found. As expected, the mortality of specialized hospitals has been more strongly reduced in markets that are more competitive after the introduction of the policy reform in comparison with medium and non-specialized hospitals. Irrespective of the underlying market structure variable, non-specialized hospitals have not changed their quality as a response to the increased competition. Turning to the DiD effects separated by the ownership form, the results show that the same result holds for public hospitals. In contrast, non-profit and private for-profit hospitals obtain positive reform effects on their quality. However, the DiD effect is not significant for private for-profit hospitals if the predicted HHI is considered to measure the market structure.

**Table 4:** Fixed effects difference-in-differences estimates of the effects of the policy reform on hospital quality moderated by ownership and specialization

	HHI				Number of hospitals	
	Actual		Predicted			
	(${\mathrm{HHI}}_{it}$)		(${\widehat{\mathrm{HHI}}}_{it}$)		(${\#\mathrm{hosp}}_{it}$)	
Model	4	5	6	7	8	9
Postref-year 2010						
(Low-spec)	0.06		0.06		0.13	
(Med-spec)	− 0.06		0.07		0.26	
(High-spec)	− 0.28*		− 0.29**		0.41**	
(Non-profit)		− 0.21*		− 0.18		0.16
(Private for-profit)		− 0.09		0.00		0.46**
(Public)		0.04		0.13		0.25
Market structure						
(Low-spec)	0.86**		0.37		0.02	
(Med-spec)	0.55		0.39		0.01	
(High-spec)	0.8		− 0.85		0.04	
(Non-profit)		0.58*		− 0.14		0.03
(Private for-profit)		1.07**		0.42		0.00
(Public)		0.84***		0.12		0.00
DiD						
(Low-spec)	0.01		− 0.01		0.00	
(Med-spec)	0.45*		0.16		− 0.01	
(High-spec)	0.79***		0.96***		− 0.03***	
(Non-profit)		0.40**		0.40**		− 0.02**
(Private for-profit)		0.45*		0.31		− 0.03***
(Public)		0.12		− 0.03		− 0.01
Controls						
Beds (50–150)	− 0.09	− 0.06	− 0.1	− 0.06	− 0.12	− 0.09
Beds (151–300)	0.02	0.06	0.01	0.08	− 0.01	0.02
Beds (301–600)	0.01	0.06	− 0.01	0.06	− 0.01	0.03
Reference (> 600)						
Log(*mortality*)	− 1.03	− 0.97	− 0.96	− 0.79	− 1.04	− 0.97
Log(*age*)	2.49	2.51	3.05	2.27	0.17	− 0.56
Log(*unemployment*)	0.59*	0.48	0.49	0.41	0.57*	0.53*
Log(*GDP*)	0.37	0.49	0.33	0.49	0.68	0.64
Observations	1894	1894	1894	1894	1894	1894
LOGLIKE	− 1239.6	− 1242.6	− 1241.5	− 1248.5	− 1235.2	− 1240.9
AIC	2511.2	2517.2	2515.1	2529.0	2502.5	2513.8

Significance levels: ***1%; **5%; *10%

Finally, the treatment effect is further decomposed by combining the degree of specialization and the type of ownership. The results are shown in Table 5. The highest variation of the DiD effects are found within the group of private for-profit and non-profit hospitals. While highly specialized non-profit hospitals have the strongest positive effects, private for-profit hospitals have a positive effect if they are medium specialized. Similar to the previous findings, the quality of non-specialized hospitals is not affected by the competition boost, irrespective of the ownership form, and public hospitals also do not react to the increased quality competition, irrespective of their degree of specialization.

**Table 5:** Fixed effects difference-in-differences estimates of the effects of the policy reform on hospital quality jointly moderated by specialization and ownership

	HHI		Number of hospitals
	Actual	Predicted	
	(${\mathrm{HHI}}_{it}$)	(${\widehat{\mathrm{HHI}}}_{it}$)	(${\#\mathrm{hosp}}_{it}$)
Model	10	11	12
Postref-year 2010			
(Non-profit and low-spec)	0.02	0.01	0.11
(Non-profit and med-spec)	− 0.06	− 0.01	0.06
(Non-profit and high-spec)	− 0.39**	− 0.40**	0.39*
(Private and low-spec)	0.00	0.01	0.13
(Private and med-spec)	− 0.08	− 0.09	0.46**
(Private and high-spec)	− 0.10	0.05	0.70***
(Public and low-spec)	0.16	0.14	0.18
(Public and med-spec)	0.13	0.50**	0.56**
(Public and high-spec)	0.01	− 0.20	0.23
Market structure			
(Non-profit and low-spec)	0.60	0.16	0.08*
(Non-profit and med-spec)	0.76	0.44	0.00
(Non-profit and high-spec)	0.52	− 0.69	0.11
(Private and low-spec)	1.05**	0.40	− 0.05*
(Private and med-spec)	0.49	0.17	0.10
(Private and high-spec)	1.63*	0.45	− 0.02
(Public and low-spec)	0.80**	0.40	− 0.04
(Public and med-spec)	0.72	0.30	0.10
(Public and high-spec)	− 0.15	− 2.82*	− 0.06
DiD			
(Non-profit and low-spec)	0.06	0.06	0.00
(Non-profit and med-spec)	0.11	− 0.02	0.00
(Non-profit and high-spec)	1.02***	1.29***	− 0.04***
(Private and low-spec)	0.09	0.00	0.00
(Private and med-spec)	0.64**	0.70*	− 0.04***
(Private and high-spec)	0.75	0.49	− 0.04**
(Public and low-spec)	− 0.08	− 0.07	0.00
(Public and med-spec)	0.51	− 0.25	− 0.01
(Public and high-spec)	0.08	0.50	− 0.02
Controls			
Beds (50–150)	− 0.12	− 0.15	− 0.15
Beds (151–300)	− 0.02	− 0.07	− 0.06
Beds (301–600)	0.00	− 0.05	− 0.02
Reference (> 600)			
Log(*mortality*)	− 0.82	− 0.73	− 0.76
Log(*age*)	0.20	0.82	− 2.37
Log(*unemployment*)	0.59*	0.47	0.57*
Log(*GDP*)	0.56	0.51	0.69
Observations	1894	1894	1894
LOGLIKE	− 1214.7	− 1213.1	− 1203.5
AIC	2497.5	2494.1	2474.9

Significance levels: ***1%; **5%; *10%

To summarize, the homogenous positive effect of the increased competition through the introduction of PR on hospital quality can be decomposed to the groups of highly specialized non-profit hospitals and private for-profit hospitals with a medium degree of specialization.

#### Robustness checks

To enhance the robustness of the causal reform effect and to minimize risks of biased estimation, we subject our DiD analysis to a series of sensitivity analyses. Some of the results are shown in tables 6, 7 and 8 in the ESM Appendix. First, we further inspect whether the parallel trend assumption holds. For this purpose, we estimated the DiD effect based on a sample of hospitals that are balanced on the pre-intervention outcome (i.e., quality in 2006) across the different groups with high, medium and low competition by means of entropy balancing [60]. The results support the parallel trend assumption, since estimated coefficients remain very similar to the estimations based on non-balanced data. Secondly, we consider a wider market definition (30 km). The results are also in line with the effects presented above. Third, we test the sustainability of the policy reform effect by considering 2012 as the post-reform year. Some of the estimators have different signs as before and none is significant, irrespective of the underlying measure of the market structure. This finding might suggest that the positive policy reform effect on hospital quality does not last. However, this wider time horizon might also increase the likelihood for confounding factors.

## Discussion

This study is the first attempt to examine the causal effect of increased competitive pressures through the introduction of public reporting on the quality of hospital care in Germany. The health policy reform to release quality performance data through PR portals in 2008 serves as an intervention for a DiD design. A homogenous effect over all hospitals of competition on quality is found. This effect is mainly driven by the response of highly specialized non-profit hospitals and medium specialized private for-profit hospitals.

In price-regulated markets, the increased transparency between hospitals should result in a fair competition for the best quality [8, 32], since firms compete for consumers on non-price dimensions such as quality [19]. However, they will increase quality to gain market share only as long as the price is above marginal cost [20]. Our results underline the heterogeneous financial situation of hospitals in Germany; some private for-profit hospitals that are more specialized with a narrower service offering, as opposed to public or non-profit hospitals with a broad range of services, can realize profits under the prospective fee-for-service payment scheme [61, 62]. Our results suggest that non-specialized hospitals, which are crucial for local emergency and acute care because of the broad variety of provided services, might not be systematically able to increase their quality in response to the competitive pressure released by PR. Instead, medium private for-profit and highly specialized non-profit hospitals realize the highest quality improvement effects.

An explanation for this difference might be provided by the distinct necessities and opportunities of the various hospital types to enhance their quality. The different ownership types and degrees of specialization might determine the hospitals’ flexibility and resources to maintain their competitive position. International (theoretical and empirical) literature suggests that non-specialized, public hospitals have in general a lower profit orientation [62, 63], limited financial resources [64–66], and a more complex and therefore rigid organizational structure, which hinder a flexible response to competitor activities [67]. Moreover, public hospitals are under pressure to serve the public by providing a broad supply of health care (see the lower degree of specialization of public hospitals in Table 1) and by avoiding situations of undersupply rather than to simply maximize efficiencies. Similarly, health care in rural areas is mostly provided by only a few hospitals that offer traditionally a broad variety of services while holding enough reserve capacities. As a consequence, public, less-specialized, more rural hospitals face more likely situations of excess capacity resulting in inefficiently high cost and negative margins as observed, e.g., in Switzerland by Widmer et al. [36]. This could serve as an explanation that these hospitals might not be able or have fewer financial incentives to invest in quality improvements as a response to the increased competitive pressure induced by PR.

In turn, managers of specialized hospitals might be more confident about the assessment of the impact of the competitors’ quality improvements on their own competitive position in comparison to non-specialized hospitals. Moreover, if needed, they can more easily increase the quality of care, because of a less complex organizational structure. Based on international findings, specialized hospitals have more organizational focus to adjust volume and structure of their services to minimize demand uncertainties [36], providing higher slack resources [64, 65] and positive margins that can be both used for enhancing quality improving activities [20, 67]. However, specialization might also be a promising tool to attract particular types of patients (cream skimming) to reduce competition faced in the hospitals’ specialist treatment area [68]. This might be an explanation for the lower policy reform effect for the highly specialized private for-profit hospitals. This supposition is supported by evidence from Australia [69] and Italy [70] that private for-profit hospitals are involved in cream skimming at a higher rate than public and non-profit hospitals.

Although we found a homogenous policy reform effect, there are several structural reasons that might limit quality competition in Germany despite legally free patient choice of hospitals. Hospitals might only invest to attract patients if they are willing to travel further for hospitals with higher quality of care than in the nearby area. In Germany, health-care mobility is still more limited (as compared to the USA) [17], which can generally result in less hospital competition and more local or regional hospital markets. For instance, the findings of Avdic et al. [71] suggest that in Germany an expectant mother is only willing to travel 0.1–2.7 additional kilometers (0.2–4.5 min by car) to give birth in a hospital that has a one standard deviation higher reported quality. Although these magnitudes are larger than estimates for other health-care services in the international literature [72, 73], they rather do not provide evidence for a general health-care mobility in Germany. Moreover, next to location and associated distance, patient choice of hospitals also considers several other non-outcome quality dimensions. As shown for England and the USA, patients often choose the hospital that they have previously attended [74, 75]. They also might choose the hospital that their outpatient physician has recommended, which still plays a large role in the less consumer-oriented German health-care market. Further, they often prefer large hospitals with academic affiliation [76]. Moreover, patients can only exercise choice if they have an option between different hospitals offering the required medical service.

### Limitations

With regard to the data and methodology employed, we consider several limitations, which might impact the interpretation of the results. Due to the focus on stroke quality of care, the hospitals not treating stroke are excluded from the sample. In 2006, 538 hospitals (out of 1902 hospitals) did not treat stroke cases. A larger share of private for-profit hospitals, a higher degree of specialization and a smaller number of beds than the other hospitals characterize the non-stroke-treating hospitals. Moreover, they only treat 8.1% of all treated inpatients and have 11.9% of all beds in 2006. Therefore, we do not believe that the concentration on stroke-treating hospitals limits the representativeness of our results.

In recent years, German hospitals have increasingly implemented stroke units. These departments are specialized in the rapid treatment of patients with stroke or suspected stroke. The number of hospitals with a stroke unit increased from 37 in 2006 (2.7% of all stroke hospitals) to 144 in 2010 (10.4%). The implementation of a stroke unit could be considered as a promising tool to increase the quality of stroke treatment [77]. However, a potential confounder on the effect of PR on hospital quality cannot be ruled out. Since the estimated coefficients and significance levels remain very similar if stroke unit hospitals are dropped from the sample, we do not believe that the general spread of stroke units has a relevant impact on the results.

In Germany, the number of mergers and acquisitions have increased in the last years leading to significant consolidation of the hospital market [78] with different hospitals owned by the same entity, so-called hospital systems [46]. As a consequence, the actual concentration levels might be underestimated [45]. Since the consolidation process might not be equally distributed across the hospitals, this process might have an impact on the effect of the introduction of PR on hospital quality. However, the incentive structure of German hospitals that is focused on patient volume can also foster competition for patients among departments within a hospital [43]. Therefore, most hospital systems might not have a well-coordinated competitive strategy and rather regard each other as competitors if they are located in the same market. Moreover, hospitals of a private group typically coordinate its regional service portfolio in such a way that the individual locations are not in competition with each other, a process that is fostered by the new hospital planning approach [79]. Therefore, we assume that the adjustment of the market structure does not have an impact on our results.^2^

In this study, we measure overall hospital quality of care by the risk-adjusted 30-day mortality rate for stroke treatment. Due to the high complexity of stroke care, quality improvements are a comprehensive task affecting the whole management of the hospital. However, several studies have shown that using different quality indicators can lead to different conclusions [80]. In practice, this can also lead to an inconsistency of hospital recommendations based on German hospital report cards with detrimental consequences for its benefit for consumers searching for hospitals that most represent their individual preferences [18]. Therefore, we cannot exclude any sensitivity of the estimated reform effects due to the used measure of quality. Future research should take into account different quality indicators as, e.g., patient-reported outcome measures to address this important issue.

Finally, the outcome quality data are based only on AOK patient-level data. However, the AOK is by far the largest health insurer in Germany, with an overall market share of 35% among publicly insured patients and an even higher share of inpatient cases. This lets us assume the representativeness of the AOK outcome data.

## Conclusion

To estimate the causal effect of hospital competition on quality of care in Germany, we employ a DiD design with the public release of quality performance data through public reporting portals in 2008 as the intervention. The market structure determines the treatment intensity, since the release of performance data is expected to have higher effects in more competitive markets. A homogenous effect over all hospitals of competition on quality can be found that is mainly driven by the response of highly specialized non-profit hospitals and medium specialized private for-profit hospitals.

Related theoretical considerations have shown that an important prerequisite for a positive effect of competition on quality is that medical services for additional patients have a positive contribution margin. This might not hold for all hospitals in Germany, especially not for the non-specialized ones. Our results suggest that the intended fair quality competition among hospitals through PR might not be optimally applied. The non-specialized hospitals that are crucial for the local acute care seems not to be able to invest in quality improvements to the same extent as their specialized competitors, which can realize quality improvements at least in the short run. To enable a fair quality competition between hospitals through PR, our results might indicate that health policy could take greater account of the different conditions and environmental circumstances of the hospitals, for instance by introducing an outcome-based payment component. If hospitals receive some additional payment for top quality performance or a deduction for poor quality, then even hospitals with a negative contribution margin would have an additional incentive to improve quality.

## Supplementary Information

Below is the link to the electronic supplementary material.

Supplementary file1 (DOCX 34 KB)

## Footnotes

1 Some hospitals operate on several so-called sites that are also geographically dispersed in some cases. We consider each location that treats stroke patients as a separate entity for the analysis and define the market from the centroid around each hospital's location. However, for computing the market structure, we sum up the number of stroke patients for all locations and consider the hospitals as one hospital as proposed by Hentschker et al. [45] for taking into account the aspect of hospital system ownership. In our sample, there are ten hospitals with more than one site. If we drop these observations from the data set as a robustness check, the results remain unaffected.

2 To examine this supposition, however, we adjusted the measures of the market structure for hospital alliances. For this purpose, we identified for each hospital the membership of an alliance (or more alliances). If two or more members are within the same hospital market, we grouped the hospitals accordingly for computing the HHI (0.54) and the number of hospitals in the market (6.7). Based on these adjusted measures, the results remain qualitatively very similar. We do not assume that our alliance identification is complete; however, the proportions of hospitals without alliance membership for markets with a 15 km (73.5%) and 30 km radius (59.1%) are rather similar to that provided by Schmid and Ulrich [46].
