# Supplementary material for: Can competition improve hospital quality of care? A difference-in-differences approach to evaluate the effect of increasing quality transparency on hospital quality
Source: Eur J Health Econ. 2022 Jan 8;23(7):1229–42. doi: 10.1007/s10198-021-01423-9 (PMC9395484; doi:10.1007/s10198-021-01423-9)
Supplement: Supplementary file 1 — Supplementary file1 (DOCX 34 KB) [file 10198_2021_1423_MOESM1_ESM.docx]

Table 6: Robustness checks on DiD effects based on Actual HHI ($HHI_{it})$

| Actual HHI ($HHI_{it})$ | Balanced on outcome | | | | Market radius: 30km | | | | Posttreatment year: $T_{0}=2012$ | | | |
| --- | --- | --- | --- | --- | --- | --- | --- | --- | --- | --- | --- | --- |
| DiD: $I\left( t=T_{1} \right)\times HHI_{i,t=T_{0}}$ | 0.33* |  |  |  | 0.34* |  |  |  | -0.17 |  |  |  |
| *(low-spec)* |  | 0.02 |  |  |  | -0.02 |  |  |  | -0.20* |  |  |
| *(med-spec)* |  | 0.52* |  |  |  | 0.41 |  |  |  | -0.10 |  |  |
| *(high-spec)* |  | 0.57 |  |  |  | 0.74* |  |  |  | -0.12 |  |  |
| *(non-profit)* |  |  | 0.40 |  |  |  | 0.38 |  |  |  | -0.29 |  |
| *(private for-profit)* |  |  | 0.60* |  |  |  | 0.24 |  |  |  | 0.01 |  |
| *(public)* |  |  | 0.03 |  |  |  | -0.06 |  |  |  | -0.12 |  |
| *(non-profit & low-spec)* |  |  |  | 0.05 |  |  |  | 0.23 |  |  |  | -0.08 |
| *(non-profit & med-spec)* |  |  |  | 0.21 |  |  |  | 0.07 |  |  |  | -0.27 |
| *(non-profit & high-spec)* |  |  |  | 0.81* |  |  |  | 1.12 |  |  |  | -0.36 |
| *(private & low-spec)* |  |  |  | 0.11 |  |  |  | 0.09 |  |  |  | -0.26 |
| *(private & med-spec)* |  |  |  | 0.60* |  |  |  | 0.42 |  |  |  | 0.06 |
| *(private & high-spec)* |  |  |  | 0.99 |  |  |  | 0.30 |  |  |  | 0.13 |
| *(public & low-spec)* |  |  |  | -0.06 |  |  |  | -0.22 |  |  |  | -0.25* |
| *(public & med-spec)* |  |  |  | 0.59 |  |  |  | 0.11 |  |  |  | -0.05 |
| *(public & high-spec)* |  |  |  | -0.45 |  |  |  | -0.15 |  |  |  | 0.27 |
| observations | 1894 | 1894 | 1894 | 1894 | 1894 | 1894 | 1894 | 1894 | 1832 | 1832 | 1832 | 1832 |
| LOGLIKE | -1401.1 | -1393.2 | -1389.9 | -1362.3 | -1258.2 | -1251.3 | -1249.0 | -1228.1 | -1111.0 | -1108.3 | -1109.0 | -1099.9 |
| AIC | 2822.1 | 2818.4 | 2811.7 | 2792.5 | 2536.4 | 2534.6 | 2530.0 | 2524.1 | 2242.0 | 2248.7 | 2250.0 | 2267.9 |

The other coefficients are not shown.

Table 7: Robustness checks on DiD effects based on Predicted HHI ($\hat{HHI}_{it})$

| Predicted HHI ($\hat{HHI}_{it})$ | Balanced on outcome | | | | Market radius: 30km | | | | Posttreatment year: $T_{0}=2012$ | | | |
| --- | --- | --- | --- | --- | --- | --- | --- | --- | --- | --- | --- | --- |
| DiD: $I\left( t=T_{1} \right)\times\hat{HHI}_{i,t=T_{0}}$ | 0.35 |  |  |  | 0.35* |  |  |  | -0.15 |  |  |  |
| *(low-spec)* |  | 0.05 |  |  |  | -0.10 |  |  |  | -0.22* |  |  |
| *(med-spec)* |  | 0.06 |  |  |  | 0.45 |  |  |  | -0.19 |  |  |
| *(high-spec)* |  | 1.10** |  |  |  | 0.93** |  |  |  | 0.12 |  |  |
| *(non-profit)* |  |  | 0.48 |  |  |  | 0.41 |  |  |  | -0.13 |  |
| *(private for-profit)* |  |  | 0.42 |  |  |  | 0.26 |  |  |  | 0.04 |  |
| *(public)* |  |  | 0.01 |  |  |  | -0.11 |  |  |  | -0.23* |  |
| *(non-profit & low-spec)* |  |  |  | 0.11 |  |  |  | -0.06 |  |  |  | -0.06 |
| *(non-profit & med-spec)* |  |  |  | -0.20 |  |  |  | 0.01 |  |  |  | -0.25 |
| *(non-profit & high-spec)* |  |  |  | 1.58 |  |  |  | 1.35 |  |  |  | 0.10 |
| *(private & low-spec)* |  |  |  | 0.04 |  |  |  | 0.05 |  |  |  | -0.25 |
| *(private & med-spec)* |  |  |  | 0.89** |  |  |  | 0.77 |  |  |  | 0.14 |
| *(private & high-spec)* |  |  |  | 0.49 |  |  |  | 0.43 |  |  |  | 0.24 |
| *(public & low-spec)* |  |  |  | -0.01 |  |  |  | -0.29 |  |  |  | -0.28* |
| *(public & med-spec)* |  |  |  | -0.42 |  |  |  | -0.36 |  |  |  | -0.41 |
| *(public & high-spec)* |  |  |  | 0.78** |  |  |  | 0.69 |  |  |  | 0.25 |
| observations | 1894 | 1894 | 1894 | 1894 | 1894 | 1894 | 1894 | 1894 | 1832 | 1832 | 1832 | 1832 |
| LOGLIKE | -1489.8 | -1471.1 | -1482.0 | -1440.0 | -1261.1 | -1253.9 | -1250.7 | -1229.2 | -1111.6 | -1105.5 | -1110.0 | -1098.5 |
| AIC | 2999.5 | 2974.2 | 2995.9 | 2947.9 | 2542.3 | 2539.8 | 2533.4 | 2526.5 | 2243.1 | 2243.0 | 2251.9 | 2265.0 |

The other coefficients are not shown.

Table 8: Robustness checks on DiD effects based on Number of hospitals (${\#hosp}_{it}$)

| Number of hospitals | Balanced on outcome | | | | Market radius: 30km | | | | Posttreatment year: $T_{0}=2012$ | | | |
| --- | --- | --- | --- | --- | --- | --- | --- | --- | --- | --- | --- | --- |
| Model | 1 | 2 | 3 | 4 | 5 | 6 | 7 | 8 | 9 | 10 | 11 | 12 |
| DiD: $I\left( t=T_{1} \right)\times({\#hosp}_{i,t=T_{0}})$ | -0.02*** |  |  |  | -0.01*** |  |  |  | 0.00 |  |  |  |
| *(low-spec)* |  | -0.01 |  |  |  | 0.00 |  |  |  | 0.00 |  |  |
| *(med-spec)* |  | -0.01 |  |  |  | 0.00 |  |  |  | 0.00 |  |  |
| *(high-spec)* |  | -0.03*** |  |  |  | -0.02*** |  |  |  | 0.00 |  |  |
| *(non-profit)* |  |  | -0.02** |  |  |  | -0.01** |  |  |  | 0.01 |  |
| *(private for-profit)* |  |  | -0.04*** |  |  |  | -0.02*** |  |  |  | -0.01 |  |
| *(public)* |  |  | -0.01 |  |  |  | -0.01* |  |  |  | 0.00 |  |
| *(non-profit & low-spec)* |  |  |  | 0.00 |  |  |  | 0.00 |  |  |  | 0.01 |
| *(non-profit & med-spec)* |  |  |  | 0.00 |  |  |  | 0.00 |  |  |  | 0.01 |
| *(non-profit & high-spec)* |  |  |  | -0.04*** |  |  |  | -0.02*** |  |  |  | 0.00 |
| *(private & low-spec)* |  |  |  | 0.00 |  |  |  | 0.00 |  |  |  | 0.00 |
| *(private & med-spec)* |  |  |  | -0.04*** |  |  |  | -0.02*** |  |  |  | -0.01 |
| *(private & high-spec)* |  |  |  | -0.05** |  |  |  | -0.03*** |  |  |  | -0.01 |
| *(public & low-spec)* |  |  |  | 0.00 |  |  |  | 0.00 |  |  |  | 0.00 |
| *(public & med-spec)* |  |  |  | -0.02 |  |  |  | -0.01 |  |  |  | 0.01 |
| *(public & high-spec)* |  |  |  | 0.00 |  |  |  | -0.01* |  |  |  | -0.02 |
| controls | yes | yes | yes | yes | yes | yes | yes | yes | yes | yes | yes | yes |
| observations | 1894 | 1894 | 1894 | 1894 | 1894 | 1894 | 1894 | 1894 | 1832 | 1832 | 1832 | 1832 |
| LOGLIKE | -1414.8 | -1407.2 | -1403.9 | -1359.5 | -1244.2 | -1225.3 | -1234.8 | -1196.8 | -1112.6 | -1107.1 | -1108.3 | -1097.8 |
| AIC | 2849.6 | 2846.4 | 2839.8 | 2787.0 | 2508.5 | 2482.5 | 2501.6 | 2461.6 | 2245.3 | 2246.3 | 2248.6 | 2263.5 |

The other coefficients are not shown.
